# Questionnaire layout and wording influence prevalence and risk estimates of respiratory symptoms in a population cohort

**DOI:** 10.1111/j.1752-699X.2012.00281.x

**Published:** 2013-01

**Authors:** Linda Ekerljung, Eva Rönmark, Jan Lötvall, Göran Wennergren, Kjell Torén, Bo Lundbäck

**Affiliations:** 1 Department of Internal Medicine, Krefting Research Centre, Institute of Medicine, Sahlgrenska Academy, University of GothenburgGothenburg, Sweden; 2 Obstructive Lung Disease In Northern Sweden (OLIN) studies, Department of Medicine, Sunderby Central Hospital of NorrbottenLuleå, Sweden.; 3 Department of Public Health and Clinical Medicine/Environmental Medicine and Occupation, University of UmeåUmeå, Sweden.; 4 Department of Paediatrics, Sahlgrenska Academy, University of GothenburgGothenburg, Sweden; 5 Department of Environmental and Occupational Medicine, Institute of Medicine, Sahlgrenska Academy, University of GothenburgGothenburg, Sweden

**Keywords:** epidemiology, GA^2^LEN, kappa, OLIN, questionnaire, respiratory symptoms

## Abstract

**Objective:**

Results of epidemiological studies are greatly influenced by the chosen methodology. The study aims to investigate how two frequently used questionnaires (Qs), with partly different layout, influence the prevalence of respiratory symptoms.

**Study Design and Setting:**

A booklet containing two Qs, the Global Allergy and Asthma European Network Q and the Obstructive Lung Disease in Northern Sweden Q, was mailed to 30 000 subjects aged 16–75 years in West Sweden; 62% responded. Sixteen questions were included in the analysis: seven identical between the Qs, four different in set-up and five with the same layout but different wording. Comparisons were made using differences in proportions, observed agreement and Kappa statistics.

**Results:**

Identical questions yielded similar prevalences with high observed agreement and kappa values. Questions with different set-up or differences in wording resulted in significantly different prevalences with lower observed agreement and kappa values. In general, the use of follow-up questions, excluding subjects answering no to the initial question, resulted in 2.9–6.7% units lower prevalence.

**Conclusion:**

The question set-up has great influences on epidemiological results, and specifically questions that are set up to be excluded based on a previous no answer leads to lower prevalence compared with detached questions. Therefore, Q layout and exact wording of questions has to be carefully considered when comparing studies.

Please cite this paper as: Ekerljung L, Rönmark E, Lötvall J, Wennergren G, Torén K and Lundbäck B. Questionnaire layout and wording influence prevalence and risk estimates of respiratory symptoms in a population cohort. *Clin Respir J* 2013; 7: 53–63.

## Introduction

Postal enquiries are among the most efficient tools when assessing prevalence and risk factors of asthma and respiratory symptoms (1, 2). The prevalence of asthma has increased over the last 50–60 years and is estimated to be 7%–10% in different parts of the Western world (3–6). Data on incidence vary partly depending on different definitions of asthma and population at risk. Using similar methods, the incidence is approximately 2/1000/year in Northern Europe (7–10). When self-reported asthma by questionnaires (Qs) is validated against clinically relevant asthma, it has a high specificity and a fair, or good, sensitivity in countries with developed health-care systems (1, 2). When comparing results from epidemiological studies, it is important to take the methods and definitions used into consideration, as results are influenced by the methodology. Two main models have been used in the validation of epidemiological diagnosis of asthma: a provocation test or a clinical interview (1, 2, 11), or a combination of both methods (12).

Today, there are few Qs that are widely used. Among adults, the European Community Respiratory Health Survey Q (ECRHS-Q) (13) and the subsequent Global Allergy and Asthma European Network Q (GA^2^LEN-Q) are commonly used. Both fail to cover bronchitis or chronic obstructive pulmonary disease (COPD) in a satisfactory way. However, the Obstructive Lung Disease in Northern Sweden Q (OLIN-Q) (14) cover these aspects and has frequently been used in Sweden and northern European countries.

In 2008, a study focusing mainly on asthma was initiated in West Sweden. The initial step was a postal survey using two respiratory Qs, the GA^2^LEN-Q and the OLIN-Q, with the primary aim of updating the prevalence of asthma, respiratory symptoms and allergy (15). The aim of the present study was to investigate how these two frequently used Qs, with partly different question structure and wording, influence the prevalence of respiratory symptoms and other outcomes.

## Materials and methods

### Study area and population

The study was initiated in 2008 when 30 000 randomly selected subjects aged 16–75 years of age received a postal Q. The study was performed in the region of West Gothia in Western Sweden, including the city of Gothenburg. The study population was selected using the Swedish Population Register and was stratified by age and sex to mirror the population in West Gothia. Study design, results of prevalence and effects of late response and nonresponse have previously been published (15, 16).

### Qs

The study consisted of a booklet containing the OLIN-Q followed by the GA^2^LEN-Q. The OLIN-Q has been used in many studies in the Nordic and the Baltic countries, prominently the FinEsS (Finland, Estonia, Sweden) studies, comparative studies of airway diseases 6, 17, 18). It was developed from the British Medical Research Council Q (BMRC-Q). The OLIN-Q contains questions on asthma, rhinitis, chronic bronchitis/COPD/emphysema, respiratory symptoms, use of asthma medication and possible determinants of disease, such as smoking habit, occupation and family history of disease. The OLIN-Q and variants of it (19) have been validated against physiological variables including bronchial hyperresponsiveness (12, 20). To this Q, detailed questions about occupation, occupational exposure, socio-economic conditions and health status were added. The Swedish version of the GA^2^LEN-Q is a variant of the ECRHS-Q (13, 21) with additional questions concerning mainly rhinitis, chronic sinusitis and eczema. Questions on rhinitis and sinusitis in the GA^2^LEN-Q originate from the Allergic Rhinitis and its Impact on Asthma initiative (21).

#### Definitions

In this comparative study, 16 questions from the two Qs were analyzed. The questions were categorized into three groups based on similarity between the Qs: Group I – identical between the Qs; Group II – same question layout but not identical symptom or condition asked for; and Group III – similar wording but different layout. The questions and differences between the Qs have been summarized in Table 1. The questions belonging to group III were follow-up questions in one of the Qs, excluding subjects who did not respond to a qualifying question, but single questions in the other. Use of asthma medication and attacks of shortness of breath were follow-up questions in the GA^2^LEN-Q, while productive cough was a follow-up question in the OLIN-Q. The qualifying question for use of asthma medication and attacks of shortness of breath was ‘Have you ever had asthma’. For productive cough the qualifying question was ‘Do you usually have phlegm when coughing, or do you have phlegm in your chest, which is difficult to bring up’. Smoking was a combination of two questions in the OLIN-Q but consisted of only one question in the GA^2^LEN-Q.

**Table 1:** Questions used in the Obstructive Lung Disease in Northern Sweden questionnaire (OLIN-Q) and the Global Allergy and Asthma European Network questionnaire (GA^2^LEN-Q)

	OLIN-Q	GA^2^LEN-Q
Group I – identical between the questionnaires		
Ever asthma	Have you ever had asthma?	[Identical to OLIN-Q]
Any wheeze	Have you had wheezing or whistling in your chest at any time in the last 12 months?	[Identical to OLIN-Q]
Wheeze with breathlessness	Have you been at all breathless when the wheezing noise was present?	[Identical to OLIN-Q]
Wheeze apart from cold	Have you had this wheezing or whistling when you did not have a cold?	[Identical to OLIN-Q]
Wheeze with breathlessness apart from cold	Yes to all three out of: any wheeze, wheezing with breathlessness and wheezing apart from cold.	[Identical to OLIN-Q]
Waking with tight chest	Have you woken up with a feeling of tightness in your chest at any time in the last 12 months?	[Identical to OLIN-Q]
Respiratory reaction to ASA	Have you ever had any difficulty with your breathing within 3 hours after taking a pain killer?	[Identical to OLIN-Q]
Group II – same question set-up but not identical symptom or condition asked for		
Rhinitis	Have you now or **have you ever had allergic rhinitis** (hay fever) or **allergic eye catarrh**.	Do you have **any nasal allergies** including hay fever?
Physician-diagnosed COPD	Have you been diagnosed as having **chronic bronchitis**, COPD or **emphysema** by a doctor?	Have you been diagnosed as having chronic obstructive pulmonary disease (COPD) by a doctor?
Nasal blockage	Do you have blocking of your nose **more or less permanently**?	Has your nose been blocked for **more than 12 weeks during the last 12 months**?
Rhinorrea	Do you have a runny nose **more or less permanently**?	Have you had discoloured nasal discharge (snot) or discoloured mucus in the throat for **more than 12 weeks during the last 12 months**?
Exposed at work	Have you been **heavily** exposed to dust, gases or fumes at your work?	Have you ever held a job where you were exposed to gases, fumes or dust?
Group III – similar wording but different set-up		
Productive cough	**Follow-up question:** Do you bring up phlegm on most days during periods of at least three months?	*Do you bring up phlegm from your chest on most days for as much as three months each year?*
Ever smoker	Do you smoke? or Have you previously smoked?	*Have you ever smoked for as long as a year?*
Asthma medication	Do you currently use asthma medication (permanently or as needed)?	***Follow-up question**: Are you currently taking any asthma medication (including inhalers, aerosols or tablets) for asthma?*
Attacks of shortness of breath	Have you now or have you had asthma symptoms during the last 12 months (intermittent breathlessness or attacks of shortness of breath, the symptoms may exist simultaneously with or without cough or wheezing)?	***Follow-up question:** Have you had an attack of asthma in the last 12 months?*

Differences have been highlighted using **bold** text.

ASA, Aspirin Sensitive Asthma.

#### Statistical analyses

Statistical analyses were performed using the Statistical Package for the Social Sciences 16.0 (SPSS, Inc., Chicago, IL, USA). Comparisons were made using differences in proportions, observed agreement (OA) and the kappa coefficient. The kappa coefficient compares the level of agreement between different groups of data (22) and was interpreted using the following definitions: below 0.2, slight or poor agreement; 0.21–0.4, fair agreement; 0.41–0.6, moderate agreement; 0.61–0.8, substantial agreement; and 0.81–1, almost-perfect agreement (23). OA measures the proportion of identical answers from the two Qs. The significance of the kappa coefficient and differences in proportions were determined by the 95% confidence interval (95% CI). *Exposed to gas*, *dust or fumes at work*, and *smoking* from each of the Qs were used as an independent variable in logistic regression analyses of questions from their respective Q to obtain relative risk estimates.

## Results

**Figure 1:**
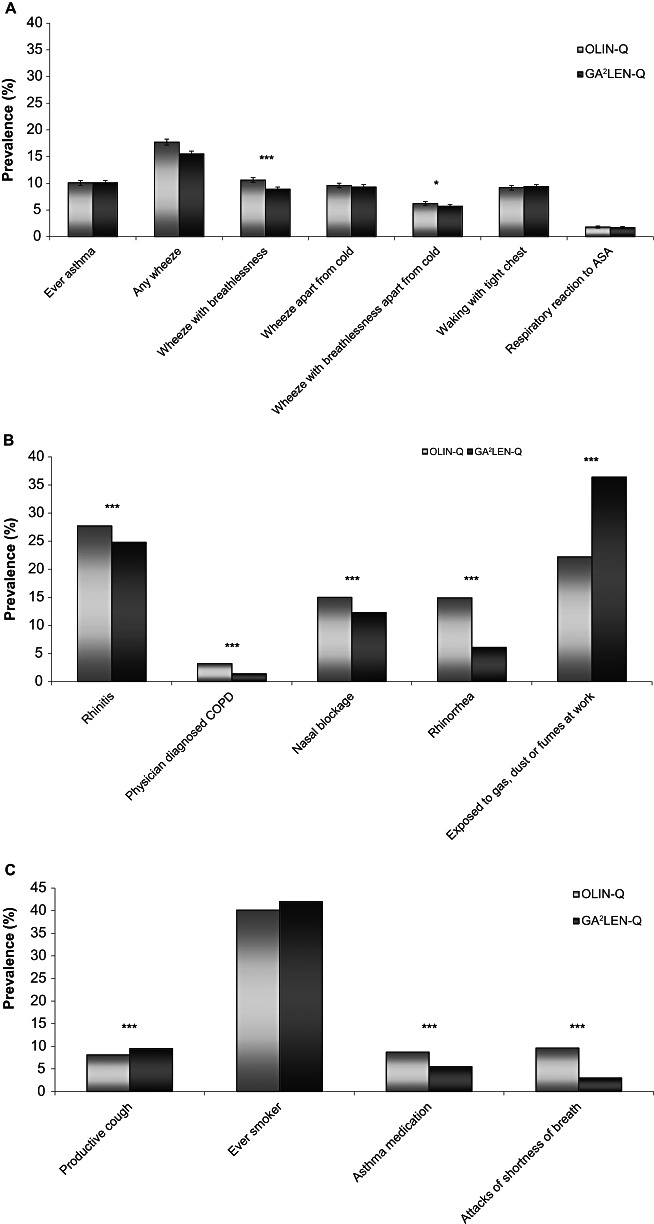
Prevalence [% with 95% confidence interval (CI)] of symptoms, disease and environmental factors by questionnaire and group. (A) Group I – identical between the questionnaires, (B) Group II – same question layout but not identical symptom or condition asked for, (C) Group III – similar wording but different layout. Bars indicate 95% CIs; significant differences between the groups are indicated as follows: *P value <0.05, **P value <0.01 and ***P value <0.001. GA^2^LEN-Q, Global Allergy and Asthma European Network Questionnaire; OLIN-Q, Obstructive Lung Disease in Northern Sweden Questionnaire. ASA, Aspirin Sensitive Asthma.

The participation in the study has previously been described in detail (15, 16). The response rate was 62% higher among women compared with men [67.4% (95% CI 66.6–68.2) vs 56.4% (95% CI 55.5–57.2)] (Table 1). Of the identical questions (group I), five yielded close to identical results, while ‘any wheeze’ 17.7% (95% CI 17.2–18.3) vs 15.5% (95% CI 15.0-16-0) and ‘wheezing with breathlessness’ 10.6% (95% CI 10.2–11.1) vs 8.9% (95% CI 8.5–9.3) were significantly different (Fig. 1A).

All five questions with similar wording but somewhat different definition (group II) had significantly different prevalences in the two Qs (Fig. 1B). Prevalence of reported ‘physician-diagnosed COPD’ was 3.2% (95% CI 2.9–3.4) using the OLIN-Q vs 1.4% (95% CI 1.2–1.6) using GA^2^LEN-Q and ‘rhinorrhea’ was 14.9% (95% CI 14.4–15.5) vs 6.1% (95% CI 5.8–6.5).

With the exception of ‘ever smoking’, all questions with a different layout between the Qs (group III) yielded significantly different results (Fig. 1C). A layout where the question was a resulting question of a previous answer (excluding subjects who had answered no to a qualifying question) yielded lower prevalence compared with a single question. Prevalent ‘use of asthma medication’ was 8.7% (95% CI 8.3–9.1) according to the OLIN-Q vs 5.5% (95% CI 5.2–5.9) in the GA^2^LEN-Q and attacks of shortness of breath 9.6% (95% CI 9.2–10.0) vs 3.0% (95% CI 2.7–3.2).

The differences in prevalence were similar in both men and women irrespective of wording and layout (Table 2). The questions had response rates ranging from 88.2% to 99.5%; all but one had response rates above 95%.

**Table 2:** Prevalence (%) and absolute difference between questionnaires (% with 95% CI) of symptoms, diseases and environmental factors by gender

	Men		
	
	OLIN	GA^2^LEN	Absolute difference (95% CI)
Group I – identical between questionnaires			
Ever asthma	8.9	8.9	0.0 (−0.008–0.009)
Any wheeze	16.3	14.3	2.0 (0.009–0.031)
Wheeze with breathlessness	9.2	7.6	1.6 (0.008–0.025)
Wheeze apart from cold	8.9	8.9	0.0 (−0.008–0.009)
Wheeze with breathlessness apart from cold	5.6	5.1	0.5 (−0.003–0.011)
Waking with tight chest	7.9	8.1	−0.2 (−0.010–0.006)
Respiratory reaction to ASA	1.3	1.2	0.1 (−0.003–0.004)
Group II – same question layout but not identical symptom asked for			
Rhinitis	26.6	24.1	2.5 (0.012–0.039)
Physician-diagnosed COPD	2.5	1.4	1.1 (0.008–0.016)
Nasal blockage	15.5	12.8	2.7 (0.016–0.037)
Rhinorrhea	14.6	6.2	8.4 (0.074–0.094)
Exposed at work	32.2	52.2	−20 (−0.214– −0.185)
Group III – similar wording but different set-up			
Productive cough	8.3	10.1	−1.8 (−0.027– −0.009)
Ever smoker	39.4	41.7	−2.3 (−0.025–0.005)
Asthma medication	6.9	4.7	2.2 (0.015–0.029)
Attacks of shortness of breath	7.6	2.4	5.2 (0.045–0.058)

	Women			
Group I – identical between questionnaires			
Ever asthma	11.0	11.1	−0.1 (−1.017–0.770)
Any wheeze	18.9	16.5	2.4 (0.013–0.035)
Wheeze with breathlessness	11.8	9.9	1.9 (0.010–0.028)
Wheeze apart from cold	10.0	9.7	0.3 (−0.004–0.013)
Wheeze with breathlessness apart from cold	6.8	6.2	0.6 (−0.001–0.013)
Waking with tight chest	10.2	10.4	−0.2 (−0.011–0.006)
Respiratory reaction to ASA	2.2	2.1	0.1 (−0.003–0.005)
Group II – same question layout but not identical symptom asked for			
Rhinitis	28.5	25.3	3.2 (0.020–0.045)
Physician-diagnosed COPD	3.7	1.5	2.2 (0.018–0.026)
Nasal blockage	14.7	11.8	2.9 (0.019–0.038)
Rhinorrhea	15.2	6.1	9.1 (0.082–0.100)
Exposed at work	13.8	23.4	−9.6 (−0.106– −0.085)
Group III – similar wording but different set-up			
Productive cough	7.9	9.1	−1.2 (−0.019– −0.003)
Ever smoker	40.7	42.3	−1.6 (−0.017–0.010)
Asthma medication	10.2	6.3	3.9 (0.031–0.046)
Attacks of shortness of breath	11.3	3.5	7.8 (0.071–0.086)

ASA, Aspirin Sensitive Asthma; CI, confidence interval; COPD, chronic obstructive pulmonary disease.

Two of the investigated questions concerned exposure to potential risk factors. One of these, ‘ever smoking’, had a prevalence of 40.1% in the OLIN-Q vs 42.0% in the GA^2^LEN-Q (Fig. 1C), while ‘exposed to gas dust or fumes at work’ was reported by 22.2% vs 36.4% (Fig. 1B).

**Figure 2:**
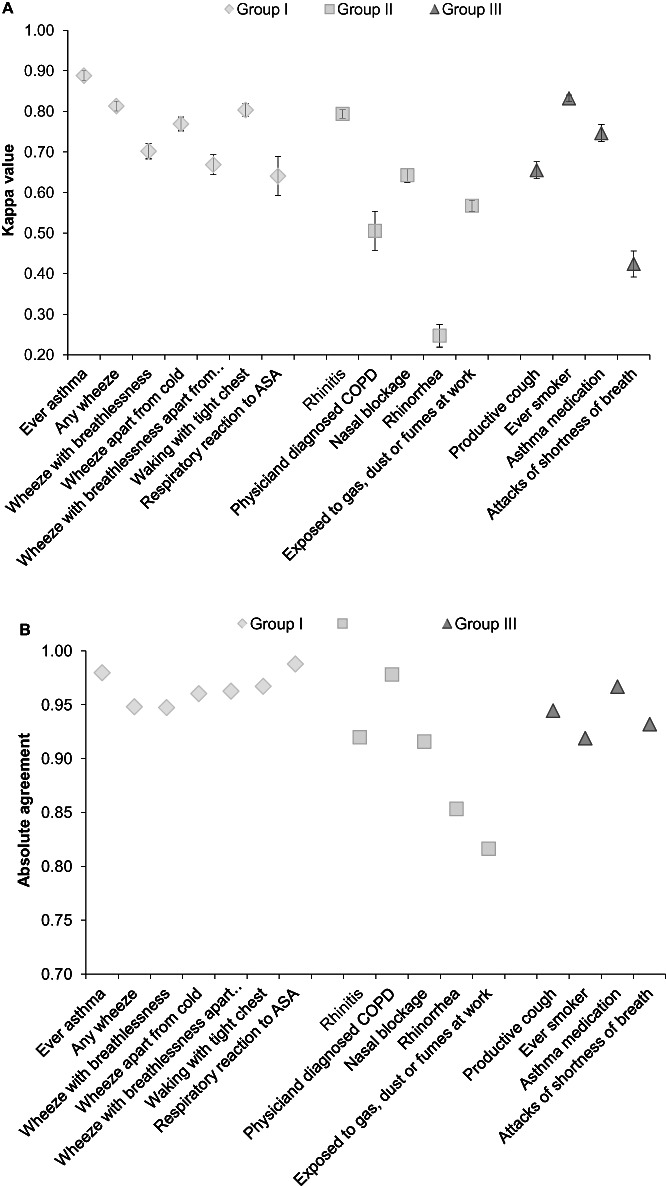
Kappa value and observed agreement with 95% confidence interval for respiratory symptoms, diseases and environmental factors. (A) Kappa values, (B) observed agreement. ASA, Aspirin Sensitive Asthma; COPD, chronic obstructive pulmonary disease.

All questions in group I had a kappa coefficient that indicated substantial or almost-perfect agreement (kappa value 0.64–0.88, Fig. 2A). ‘Ever asthma’ had the best agreement (kappa 0.89, 95% CI 0.88–0.90) followed by ‘any wheeze’ (kappa 0.81, 95% CI 0.80–0.83). In group II, two of the questions had kappa values above 0.6, while ‘physician-diagnosed COPD’ (kappa 0.51, 95% CI 0.51–0.51) and, in particular, ‘rhinorrhea’ (kappa 0.24, 95% CI 0.24–0.24) had low agreement between the Qs. The highest kappa value was found for ‘rhinitis’ with a kappa of 0.79 (95% CI 0.79–0.79), which was higher than for most of the symptoms in group I. In group III, ‘productive cough’ had a kappa value of 0.66 (95% CI 0.63–0.68), ‘ever smoker’ 0.83 (95% CI 0.82–0.084) and ‘asthma medication’ 0.75 (95% CI 0.73–0.77), thus being higher than most kappa values in group II. Only attacks of shortness of breath had a poorer degree of agreement.

The proportion of identical answers from corresponding questions in the two Qs was in general very high, with OA above 0.92 (Fig. 2B). Only ‘rhinorrhea’ (OA 0.85) and ‘exposed to gas dust or fumes at work’ (OA 0.82) had a somewhat lower proportion of identical answers. There were no differences in reliability between different subpopulations, such as high vs low education, non smoking vs smoking and men vs women.

**Figure 3:**
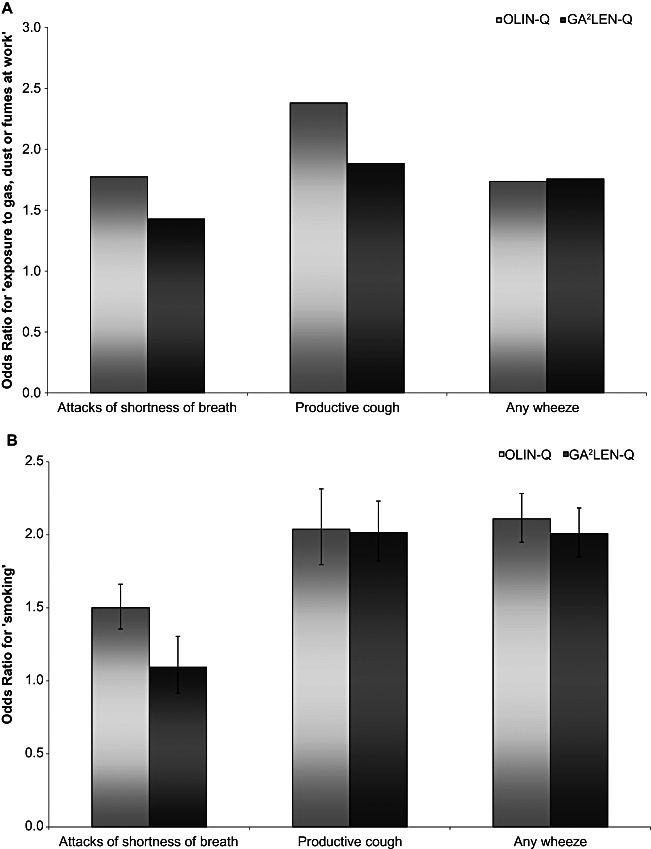
Risk-factor comparisons for the Obstructive Lung Disease in Northern Sweden Questionnaire (OLIN-Q) and Global Allergy and Asthma European Network Questionnaire (GA^2^LEN-Q). (A) ‘Exposure to gas, dust or fumes at work’ as the independent variable. (B) ‘Smoking’ as the independent variable. Data is presented as odds ratios with 95% confidence iinterval calculated using logistic regression.

A risk-factor analysis revealed no differences regarding relative risk estimates for ‘any wheeze last 12 months’ using the two Qs for either of the investigated independent variables ‘exposed to gas dust or fumes at work’ (Fig. 3A) or ‘smoking’ (Fig. 3B). Both ‘exposed to gas dust or fumes at work’ and ‘smoking’ were stronger risk factors in the OLIN-Q for ‘attacks of shortness of breath’, while ‘exposed to gas dust or fumes at work’ was a stronger risk factor also for ‘productive cough’ in the OLIN-Q. There were no differences between the two Qs in odds ratios for ‘smoking’ as a risk factor for ‘productive cough’.

## Discussion

This study compares the GA^2^LEN-Q, which can be regarded as a variant of the ECRHS-Q (13), with the OLIN-Q, a Q used mainly used Northern European countries 15, 18, 20, 24). Similar estimates of prevalence of symptoms and diseases were found with a few important exceptions. OA was above 0.9, and kappa values indicated substantial or almost-perfect agreement in most cases. In general, a slightly lower prevalence was found in the GA^2^LEN-Q compared with the OLIN-Q. Risk estimates were dependent on the prevalence of the independent variables and generally higher with the OLIN-Q as a result of wording and the design of questions about exposure. Questions about nasal symptoms were more detailed in the GA^2^LEN-Q, while questions about bronchitis were more detailed in the OLIN-Q. Questions regarding symptoms common in asthma were similar or identical in the two Qs.

Nonresponse is an issue for all epidemiological studies as it might introduce bias. A nonresponse study has been performed on the study sample, and nonresponders were more likely to be male, younger, living in Gothenburg and smokers. However, this did not influence the prevalence or risk estimates (16). The large study sample, representative of the general population in the study area, ensures the validity of the study.

Results from Qs are dependent on several factors. Self-administered Qs results in higher prevalences than structured interviews (25–27); translations create variability (28, 29), particularly the translation of ‘wheeze’ and responses to self-administered Qs before and after a physical demonstration of asthma symptoms results in divergent results with kappa statistics below 0.4 (30). The agreement may also vary with smoking habits and educational level (28). In two studies, the kappa statistics has decreased with increasing educational (28) and social-economical (30) status. However, in the Norwegian study (28), the agreement increased with increasing educational level. An overview of Q comparisons can be found in Table 3.

**Table 3:** Observed agreement (OA) and kappa for asthma, attacks of shortness of breath, wheezing and sputum production in previous studies

	Asthma		Attacks of shortness of breath		Wheezing		Sputum production	
				
Study	OA	Kappa	OA	Kappa	OA	Kappa	OA	Kappa
Comparisons of different questionnaires								
Arizona-Q vs NHLI-Q (26)	0.79	–	0.93	–	0.90	–	0.84	–
Arizona-Q vs BMRC-Q (26)	–	–	0.89	–	0.88	–	0.85	–
BMMRC-Q vs IUATLD-Q (31)	–	0.90	–	–	–	0.72	–	–
BMRC-Q vs NR-Q (28)	–	–	–	–	0.91	0.73	0.88	0.51
BMRC-Q vs NHLI-Q†(27)	–	–	–	–	0.73	–	0.80	–
BMRC-Q vs ATS-Q (25)	0.99	–	–	–	–	–	–	–
Comparisons of the same questionnaire								
ECRHS-Q‡(30)	–	–	0.69*	–	0.67	–	–	–
EPIC-Potsdam study†(34)	–	0.72	–	–	–	–	–	–
IUATLD-Q§(29)	–	0.70–1.00	–	0.40–0.46	–	0.73–0.95	–	–
ECSC-Q, four occasions (2)	0.96	–	–	–	0.72	–	–	–
BMMRC-Q (2)	–	0.66	–	–	–	0.66	–	–
ATS-DLD†(2)	0.96	–	–	–	0.76	–	–	–

* Attacks of shortness of breath upon exercise.

† Interview vs self-completed.

‡ Before and after demonstration of asthma symptoms.

§ Comparisons made in four countries.

ATS-Q, American Thoracic Society Questionnaire; BMRC-Q, British Medical Research Council Questionnaire; BMMRC-Q, British Modified Medical Research Council Questionnaire; ECRHS-Q, European Community Respiratory Health Survey Questionnaire; ECSC-Q, European Community for Coal and Steel Questionnaire; EPIC, European Prospective Investigation into Cancer and Nutrition; IUATLD-Q, International Union Against Tuberculosis and Lung Diseases Questionnaire; NHLI-Q, National Heart and Lung Institute Questionnaire; NR-Q, Norwegian Respiratory Questionnaire.

The two Qs compared in this study have slightly different foci, which influence how the data can be analyzed, and have an impact on prevalence and relative risk estimates. The GA^2^LEN-Q provides more information about rhinitis and eczema compared with the OLIN-Q. The OLIN-Q provides a more thorough description of bronchitis symptoms. It also detects asthma-like symptoms not only among subjects with asthma but in the general population. The GA^2^LEN-Q excludes all nonasthmatics to some questions as it includes qualifying questions and will therefore only report the prevalence of use of asthma medication and attacks of shortness of breath among asthmatics and cannot give an estimate of prevalence in the general population. The slight difference in target population for some questions makes comparison of prevalence estimates more difficult. To detect accurate prevalence of symptoms common in asthma, we suggest that all questions should be answered by all participants.

In line with previous comparisons of Qs regarding prevalence 25, 31, 32), identical questions and wordings yielded similar estimates of prevalence in our study. Because of the high power of the study comprising of 18 087 participants, several questions resulted in statistically significant differences despite small differences in prevalence, differences that are likely to be of limited clinical relevance. For questions with a similar layout but containing different conditions, the prevalences differed to a higher degree between Qs. Despite covering the same symptom category, significant differences in prevalence have been previously observed even for questions that appear similarly worded (28).

Despite differences in prevalence outcomes, the OA for all questions was very high, including questions with different wording and different layout. The OA in our study was greater than in many previous validation studies, including the National Heart and Lung Institute Q vs the BMRC-Q, and the International Union Against Tuberculosis and Lung Diseases Q (IUATLD-Q) against the BMRC-Q (31).

The kappa values in our study for identical questions all showed almost-perfect (>0.8) or substantial (0.6–0.8) agreement. Even though these kappa values varied from 0.64 to 0.88, we had anticipated even closer levels of agreement. However, the kappa values in the current survey are similar or better compared with studies where the same Q had been distributed twice to the same subjects a few months apart (2), and also when compared with repeatability of the IUATLD-Q (29) and the comparison of the IUATLD-Q vs the BMRC-Q (31).

In order to illustrate how the way a question is asked influence the relative risk estimates in an epidemiological study, risk-factor analyses were performed using questions from different groups. The calculations of relative risk estimates tended to yield higher odds ratios when using the OLIN-Q. If the prevalence is high, and the kappa and OA are satisfactory for the symptom in question, the risk-factor patterns will be similar. However, if the symptoms have been defined differently and hence have different prevalence outcomes, the risk-factor patterns are more divergent.

It is known that self-reports are influenced by wording, format and context (33). The precise wording and tempus of a question can thus increase or decrease the probability of a positive response, with more precise questions rendering lower prevalence and questions including words such as ‘have you ever’ rendering higher prevalence than questions using ‘have you now’ wording. This phenomenon can be seen in the ‘rhinorrhea’ and ‘nasal blockage’ questions, which are more precise in the GA^2^LEN-Q. Differences may also be explained by factors other than wording and layout of the questions. The two Qs together amounted to 74 questions. Although the subjects were not specifically asked to complete the Qs in a specific order, it can be assumed that a vast majority answered the OLIN-Q first as it was placed first in the booklet that contained the Qs. This could have an influence on answers to questions placed further into the booklet. Furthermore, answering questions about symptoms prior to questions about reactions to environmental conditions might also make the subject more aware of their disease and therefore more prone toward a positive response.

## Conclusions

Identical questions yielded close to identical results regarding prevalence and had high levels of OAs and kappa values. Both Qs result in similar prevalence, primarily of lower respiratory symptoms. Different wording and different layout had a substantial influence on the estimated prevalence and risk-factor patterns and must be taken into account when comparisons between different studies are performed. An important aspect to consider when epidemiological methods to quantify the prevalence of asthma and symptoms common in asthma are evaluated is to remember that we lack an exact definition of the disease and cannot be certain which method correctly mirrors the truth. The importance of presenting, or referencing, the exact questions in any Q-based survey, not only in the respiratory field, cannot be emphasized strongly enough.
